# Downregulation of Uncoupling Protein 2(UCP2) Mediated by MicroRNA-762 Confers Cardioprotection and Participates in the Regulation of Dynamic Mitochondrial Homeostasis of Dynamin Related Protein1 (DRP1) After Myocardial Infarction in Mice

**DOI:** 10.3389/fcvm.2021.764064

**Published:** 2022-02-24

**Authors:** Dehui Liu, Shangrong Zou, Guangnan Li, Qiyu Zhang, Chunlin Chen, Cuizhi Li, Huafeng Song, Shaoxian Chen, Jiawen Wang, Yueheng Wu, Youbin Liu

**Affiliations:** ^1^Department of Cardiology, Guangzhou Eighth People's Hospital, Guangzhou Medical University, Guangzhou, China; ^2^Department of Pharmacy, Guangzhou Eighth People's Hospital, Guangzhou Medical University, Guangzhou, China; ^3^Department of Cardiology, The Fourth Affiliated Hospital of Harbin Medical University, Harbin, China; ^4^Guangdong Provincial Key Laboratory of South China Structural Heart Disease, Guangdong Cardiovascular Institute, Guangdong Provincial People's Hospital, Guangzhou, China; ^5^School of Forensic Medicine, Guizhou Medical University, Guiyang, China

**Keywords:** myocardial infarction, UCP2, DRP1, mitochondrial fission, microRNA

## Abstract

Acute myocardial infarction (MI) is one of the leading causes of death in the world, and its pathophysiological mechanisms have not been fully elucidated. The purpose of this study was to investigate the role and mechanism of uncoupling protein 2 (UCP2) after MI in mouse heart. Here, we examined the expression and role of UCP2 in mouse heart 4 weeks after MI. The expression of UCP2 was detected by RT-PCR and western blotting. Cardiac function, myocardial fibrosis, and cardiomyocyte apoptosis were assessed by echocardiography and immunohistochemistry. Phosphatase dynamin-related protein1 (*P*-DRP1) and myocardial fibrosis-related proteins were measured. Cardiomyocytes were exposed to hypoxia for 6 h to mimic the model of MI. Mdivi, an inhibitor of *P*-DRP1, was used to inhibit DRP1-dependent mitochondrial fission. Mitochondrial superoxide, membrane potential, oxygen consumption rate, and cardiomyocyte apoptosis were detected after hypoxia. It is shown mitochondrial superoxide, membrane potential, oxygen consumption rate, and cardiomyocyte apoptosis were dependent on the level of *P*-DRP1. UCP2 overexpression reduced cardiomyocyte apoptosis (fibrosis), improved cardiac function and inhibit the phosphorylation of DRP1 and the ratio of *P*-DRP1/DRP1. However, inhibition of DRP1 by mdivi did not further reduce cell apoptosis rate and cardiac function in UCP2 overexpression group. In addition, bioinformatics analysis, luciferase activity, and western blot assay proved UCP2 was a direct target gene of microRNA-762, a up-regulated microRNA after MI. In conclusion, UCP2 plays a protective role after MI and the mechanism is involved in microRNA-762 upstream and DRP1-dependent mitochondrial fission downstream.

## Introduction

Acute myocardial infarction (MI) caused by coronary heart disease (CHD) is very common in clinical practice and is associated with high morbidity and mortality (1). MI leads to cardiac dysfunction, arrhythmias and abnormal remodeling of the ventricles. The pathophysiological process of this disease is complex and its mechanisms have not been entirely elucidated (2). A deeper understanding of the pathophysiological mechanisms is necessary to better treat this disease.

Uncoupling protein 2 (UCP2) is a mitochondrial transport protein, which is increasingly recognized as an important molecule in the defense of various cardiovascular diseases such as atherosclerosis, coronary heart disease, heart failure and hypertension (3–7). The protective effect of UCP2 on atherosclerosis has been studied, and its mechanism may be related to macrophage metabolism and thermogenesis (8). It has been reported that transgenic mice with UCP2 are protected against salt-induced hypertension (9). There are also some pieces of evidence show UCP2 has a significant protective effect on ischemia/reperfusion injury (10). However, the roles of UCP2 in the heart have not yet been fully elucidated. Even in the same disease, UCP2 may play different roles. For example, concerning CHD, some studies demonstrated that diabetic patients after MI cohort with the UCP2-866A allele have poorer survival (11, 12). However, another study showed the UCP2-866A allele is associated with reduced risk of CHD in type 2 diabetic men in a 6-year prospective study (13). Therefore, there is an urgent need to establish the roles of UCP2 in CHD, and this study aims to explore the role of UCP2 in mouse heart after MI.

In this study, we found the expression of UCP2 is down-regulated by miR-762 and UCP2 has a protective role in cardiomyocyte apoptosis and fibrosis, and improve cardiac function after MI. The protective mechanism of UCP2 on the process is involved in DRP1-dependent mitochondrial fission. UCP2 may be a new therapeutic target for patients after MI.

## Materials and Methods

### Materials

The chemical substances, antibodies, and reagents used herein were obtained from various sources. Dulbecco's modified Eagle medium (DMEM), phosphate buffer saline (PBS), Collagenase, fetal bovine serum (FBS), and Lipofectamine 3,000 were purchased from Sigma-Aldrich (Merck KGaA, Darmstadt, Germany); Mitochondrial division inhibitor 1 and MitoSOX Red Mitochondrial Superoxide Indicator were purchased from MedChemExpress(Shanghai); TRIzol reagent and mRNA qRT-PCR Sybr Green Detection Kit were purchased from Invitrogen (USA). An Annexin V-FITC, Propidium Iodide (PI) Detection Kit,m and A terminal deoxynucleotidyl transferase-mediated dUTP nick end-labeling (TUNEL) kit were purchased from BD Biosciences (New Jersey, USA); The following primary antibodies were used in this experiment: anti-GAPDH (1:100, Cell Signaling Technology, USA), anti-UCP2 (1:5,000, R&D Systems, Inc, USA), anti-MMP9 (1:1,000, R&D Systems, Inc. USA), anti-TGF-beta (1:1,000, Abcam, Cambridge, Britain), and anti-p-DRP1 (1:1,000, Cell Signaling). MTT assay (Beyotime); LDH assay (Beyotime).

### Establishment of the Mouse MI Model

The left anterior descending (LAD) coronary artery of male C57BL/6 (20–25 g) was subjected to ligation to induce MI induce MI as described previously (14). In short, pentobarbital sodium (50 mg/kg) was used to anesthetize mice intraperitoneally and mice were ventilated artificially with a rodent ventilator. After sedation, thoracotomy, and pericardiotomy were performed, followed by ligation of the beginning of the LAD coronary artery with 6-0 proline suture. The ST-segment elevation of ECG indicates the successful induction of myocardial ischemia. Mice in the sham-operated group received the same treatment but did not receive LAD coronary artery ligation. After 4 weeks, all mice were examined by echocardiography to confirm that the models of heart failure were successfully induced, and then they were euthanized by cervical dislocation so that their tissues could be used for subsequent experiments. All animal experiments were approved by the Animal Care and Use Committee of the Institute of Medicine, Guangdong Provincial People's Hospital. All animal experiments were conducted following the guidelines for the National Institutes of Health guide for the care and use of laboratory animals (NIH Publications No. 8023, revised 1978, UK).

### Masson's Trichrome Staining

Masson's trichrome staining was performed to detect collagen fibers in tissue that were fixed in paraformaldehyde (4%) and embedded in paraffin. After staining, the collagen fibers were blue, the nuclei were black, and the cytoplasm was red.

### Cell Culture and Hypoxia Treatment

Established procedures were used to isolate primary neonatal mouse cardiomyocytes (NMCMs) (15). Neonatal C57BL/6 mice (1–2 d old) were euthanized by decapitation and hearts were immediately extracted. The ventricles were quickly and finely minced, followed by digestion with 0.1% collagenase. Cell suspensions were collected, centrifuged, and then resuspended in DMEM with 100 U/ml penicillin, 10% FBS, and 100 μg/ml streptomycin. Cells were expanded under standard culture conditions (37°C in 5% CO2 and 95% O2) for 1.5 h to obtain fibroblast attachment to the culture plates. Then, the cell suspension primarily containing NMCMs was collected and plated onto culture dishes. After 24 h, NMCMs were attached and cultured in the complete medium containing 0.1 mM 5-BrdU. Cell purity was confirmed by cellular morphology (beating cells) and immunostaining. Monoclonal antibodies against GAPDH were used to identify cardiomyocytes.

To establish the hypoxia model, the culture medium was replaced with serum-free DMEM and the cells were placed in an anaerobic chamber containing 94% N2, 5% CO2, and 1% O2. In the normoxia group, cells were cultured in serum-free DMEM and placed in a normoxic incubator. The cells were subjected to hypoxia for 24 h unless indicated otherwise.

### Transfection of miR-762 Mimic or Inhibitor Into NMCMs

The cells were transfected for 24 h with 50 nM miR-762 mimic (Product Number: miR1171220024715-1-5, Ribobio, Guangzhou, China) or 100 nM miR-762 inhibitor (Product Number: MIR2171220024749-1-5, Ribobio) using lipofactamin RNAiMAX (Invitrogen, US) according to the manufacturer's protocol. Cells transfected with negative control of mimic (NCm) (Product Number: miR1N0000001-1-5, Ribobio) or negative control of inhibitor (NCi) (Product Number: miR2N0000001-1-5, Ribobio) served as controls.

### Luciferase Reporter Gene Assay

In order to construct reporter vector with miRNA-762 target site, wild-type or mutant UCP2 mRNA 3′UTR sequence was amplified by polymerase chain reaction (PCR) and cloned into pGL3-promoter construct (Promega). The wild-type or mutant UCP2 3′UTR firefly luciferase reporter gene was obtained. Myocytes were co-transfected with 80 ng wild-type or mutant UCP2 3′UTR firefly luciferase reporter gene, 40 ng Renilla luciferase reference plasmid PRL-TK, and miR-762 (final concentration, 20 nM) in each control. Luciferase activity was measured 48 h after transfection using the dual luciferase reporter assay system (Promega, USA). The final data is the ratio of firefly fluorescent value to Renilla fluorescence value.

### Quantitative Real-Time RT-PCR

To determine the expression of UCP2 mRNA, total RNA was separated from cardiac tissues and cultured cells using TRIzol reagent. NanoDrop −2,000 spectrophotometer (Thermo Fischer Scientific) was used to assess RNA quality and purity. To quantify the mRNA expression of UCP2, SYBR RT-PCR was used to synthesize cDNA and make a quantitative RT-PCR analysis. Ct (threshold cycle) values were used to quantify mRNA expression levels. GAPDH served as the internal control for mRNA. The mRNA expression levels were expressed as fold changes relative to the levels of the appropriate control samples and were determined using the 2-ΔΔCt method. The following primers were used for the experiment:

mouse UCP2:
Forward Primer
5-ATGGTTGGTTTCAAGGCCACA-3
Reverse Primer
5-TTGGCGGTATCCAGAGGGAA-3
mouse GAPDH:
Forward Primer
5-AGGTCGGTGTGAACGGATTTG-3
Reverse Primer
5-TGTAGACCATGTAGTTGAGGTCA-3

### Western Blot Analysis

The procedure was followed as in a previous study (16). Protein samples of cells or tissues were washed with PBS and lysed on ice in lysis buffer supplemented with protease inhibitors and phosphatase inhibitors. Then the samples were centrifuged at a speed of 12,000 × g for 15 min at 4°C. Protein was separated by 10% SD polyacrylamide gel electrophoresis and then transferred to PVDF membranes. These membranes were immersed in 5% non-fat dry milk in TBST with 0.1% Tween-20 for 1.5 h at room temperature, rinsed, and incubated overnight at 4°C with specific antibodies against UCP2, P-DRP1, MMP9, and TGF-ß in Tris-buffered saline (TBS) containing 0.1% Tween-20 (TBS-T). Primary antibodies were cleaned by washing the membranes three times in TBS-T and then incubated for 1 h with a horseradish peroxidase-conjugated secondary antibody (1: 1,000–2,000 dilution). Membranes were was cleaned three times with TBS-T. Then, immunopositive bands were visualized by enhanced chemiluminescence and exposed to X-ray film. The levels of protein expression were presented as fold changes relative to expression levels in the control sample.

### Flow Cytometry Analysis

To analyze the effects of treatments on cell survival, Annexin V-FITC and PI Detection Kit were used to stain the cells before flow cytometry analysis. The steps are as follows in detail: NMCM cells were collected at the logarithmic phase, digested with 0. 25% trypsin, and then washed with pre-cooled PBS 2 × 5 min. Annexin V-FITC (5 μl) and PI (5 μl) were added to a buffer with 500 μl suspended cells. The apoptosis rate was determined by flow cytometry. Total apoptosis rate (%) = early apoptosis rate + late apoptosis rate. Repeat three times for each sample.

### Cell Viability and Cell Injury Evaluation

As previously reported, the MTT assay (Beyotime) was used to determine cell (NRCMs) viability. According to the manufacturer's instructions, cell damage is assessed by determining the release of LDH from the cell using the LDH detection kit (Beyotime).

### TUNEL Assay

After MI, mice were sacrificed and the left ventricle was removed. The left ventricular myocardium was placed in a 10% formaldehyde solution and fixed for 24 h. The tissue was embedded with paraffin. Twelve slices were taken from each group, and 20- 400-fold fields were randomly selected from each section to count the number of cells in the field that were positive for staining. AI (%) = the number of positive cells in the field/the total number of cells in the field × 100%.

### OCR Assay

To assess the Oxygen Consumption Rate (OCR), NMCMs cells were obtained from the mice after hypoxia or Ad-UCP2/LacZ treatment and rinsed briefly in Seahorse XF medium supplemented with 2 mM L-glutamine, 10 mM glucose, and 1 mM sodium pyruvate. The OCR was assessed at 37°C in a Seahorse XF24 extracellular flux analyzer (Agilent) to the value effects of Ad-UCP2 or AdLacZ on OCR. Once the XF experiment was completed, cells were homogenized to determine the protein concentration for normalization. Protein concentrations were determined using a bicinchoninic acid (BCA) protein assay kit (HyClone Pierce). The production of ATP and basal respiration rate were detected by observing how the OCR changes in response to treatments that modulate mitochondrial activity.

### Measurement of Mitochondria Reactive Oxygen Species

Cells were collected in different treatment groups, washed with PBS 3 times, and then resuspended in PBS. Then, the cells and MitoSOX with appropriate concentration were placed in the cell suspension for 30 min. After 30 min, they were washed with PBS. After centrifugation, a flow cytometer (Thermo Fischer Scientific) was used to detect the fluorescence intensity of MitoSOX in 510–580 waves.

### Determination of Mitochondrial Membrane Potential

After a 24 h hypoxia treatment, NMCMs were perfused with Tyrode's solution containing 50 nM blebbistatin and 50 nM tetramethylrhodamine methyl ester (Guyana Biotech Co., Ltd. (Shanghai, China) for 30 min. Confocal images were taken at 20x magnification from multiple randomly selected regions before and after hypoxia with a Nikon A1 confocal microscope(Nikon AmericaInc., Melville, NY).

### Statistical Analysis

All data are presented as the mean ± S.D of at least three independent experiments. Differences between two groups were analyzed by Student's test and multiple comparisons were analyzed by one-way ANOVA using GraphPad Prism version 8.0 (GraphPad). *P* < 0.05 was considered statistically significant.

## Results

### The Expression of UCP2 After MI

**Figure 1:**
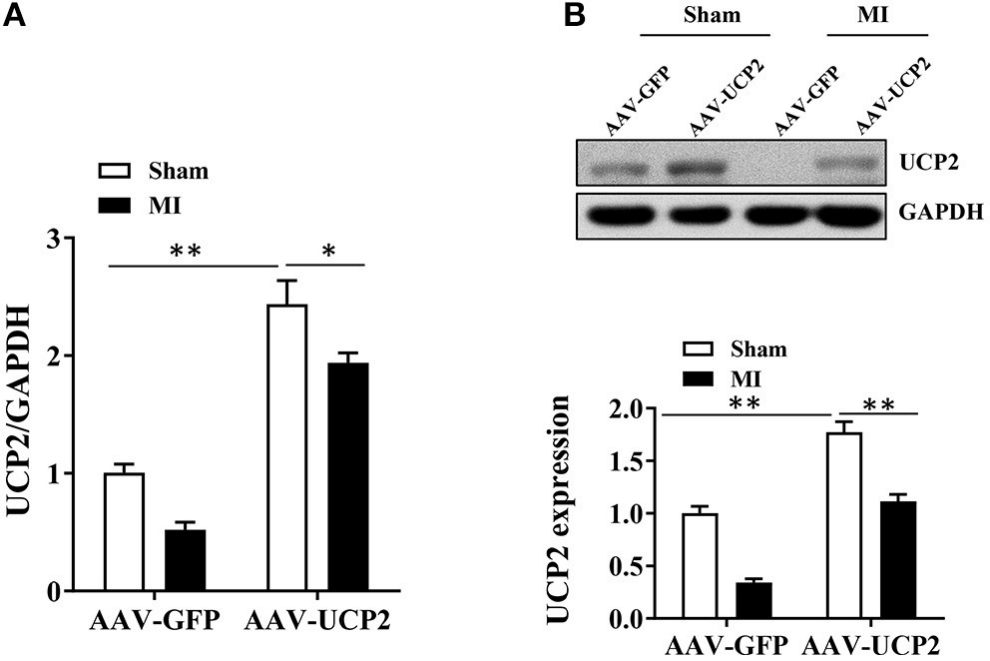
The expression of UCP2 in the infarcted marginal zone was significantly down-regulated 4 weeks after MI in mice. **(A)** qRT-PCR was performed to detect the expression of UCP2 in myocardial tissue under sham and over-expressed UCP2 condition 4 weeks after MI. (*n* = 5); **(B)** Western blotting was performed to detect the expression levels of UCP2 in myocardial tissue under sham and over-expressed UCP2 condition 4 weeks after MI. (*n* = 5); MI, myocardial infarction; *p < 0.05; **P < 0.01 compared with each other.

Previous studies have shown UCP2 is expressed in myocardial tissue (17, 18). Increasing evidence indicates the expression of UCP2 is upregulated in the ischemic myocardium (19). To explore the expression of UCP2 in the post-infarction myocardium, we measured UCP2 by PCR and western blotting in infarct border zone. It was found the expression of UCP2 decreased significantly 4 weeks after MI which is shown in the AAV-GFP group in Figure 1. To further explore the role of UCP2, we constructed the AAV-UCP2 vector. Mice were injected with this vector intravenously, and the gene and protein levels of UCP2 in the myocardial tissue were detected 4 weeks after MI. It is shown the expression of UCP2 was significantly increased in AAV-UCP2 group, which indicating a successful vector construct.

### UCP2 Improves Cardiac Function, Reduces Cardiomyocyte Apoptosis and Fibrosis After MI

**Figure 2:**
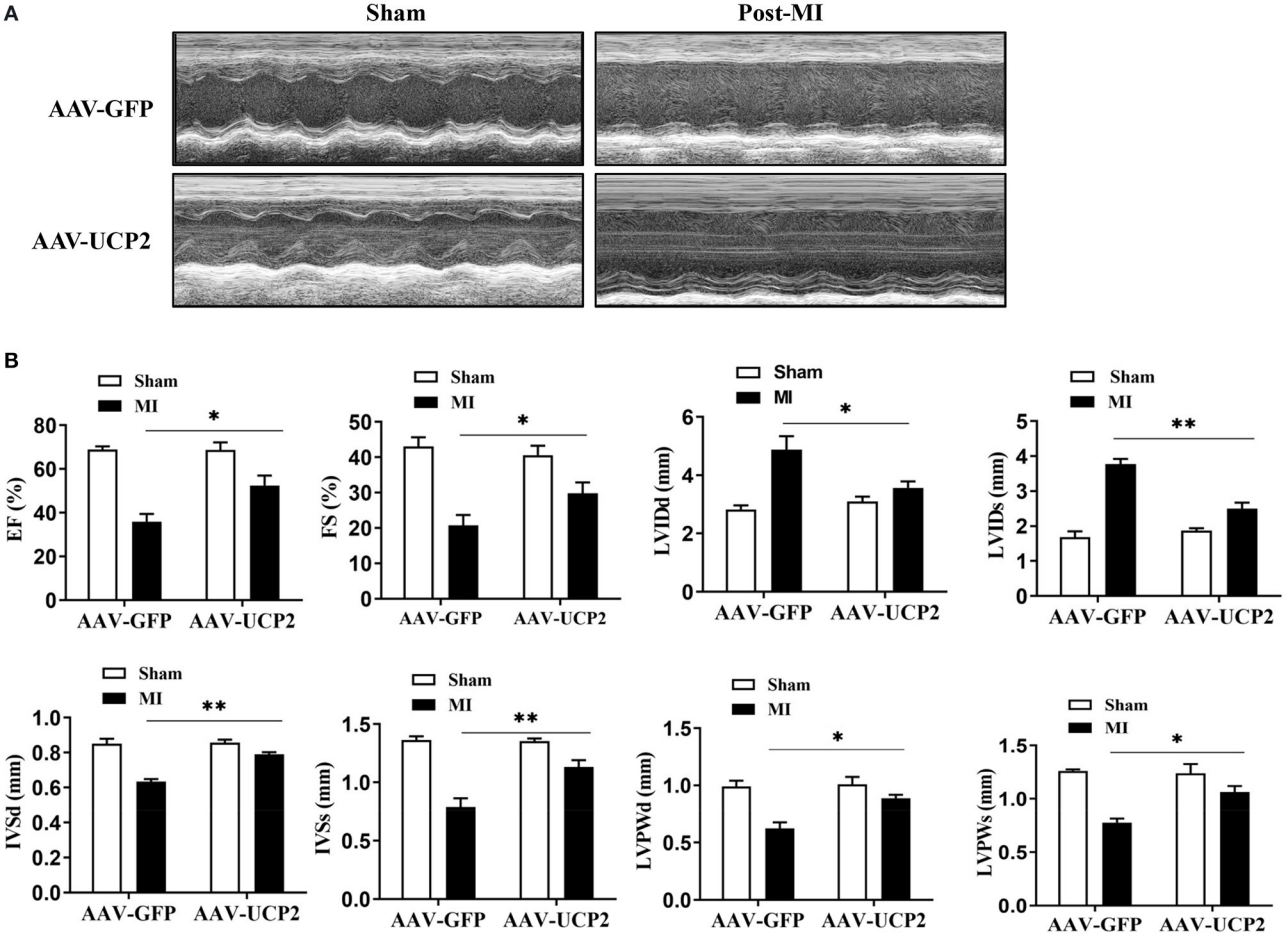
Left ventricular functional and quantitative echocardiographic analysis in sham operation and over-expressed UCP2 group 4 weeks after MI. **(A)** Two-dimensional ultrasound diagram in sham-operated group and over-expressed UCP2 group 4 weeks after MI. **(B)** Functional and quantitative echocardiography in sham-operated group and over-expressed UCP2 group 4 weeks after MI. EF, Ejection fraction; FS, Fractional shortening; LVIDd, Left ventricular end diastolic diameter; LVIDs, Left ventricular end systolic diameter; LVPWd, Left ventricular posterior wall diastolic diameter; LVPWs, Left ventricular posterior wall systolic diameter; IVSs, Interventricular septal systolic diameter; IVSd, Interventricular septal diastolic diameter; MI, Myocardial infarction; *p < 0.05, **P < 0.01 compared with each other.

**Figure 3:**
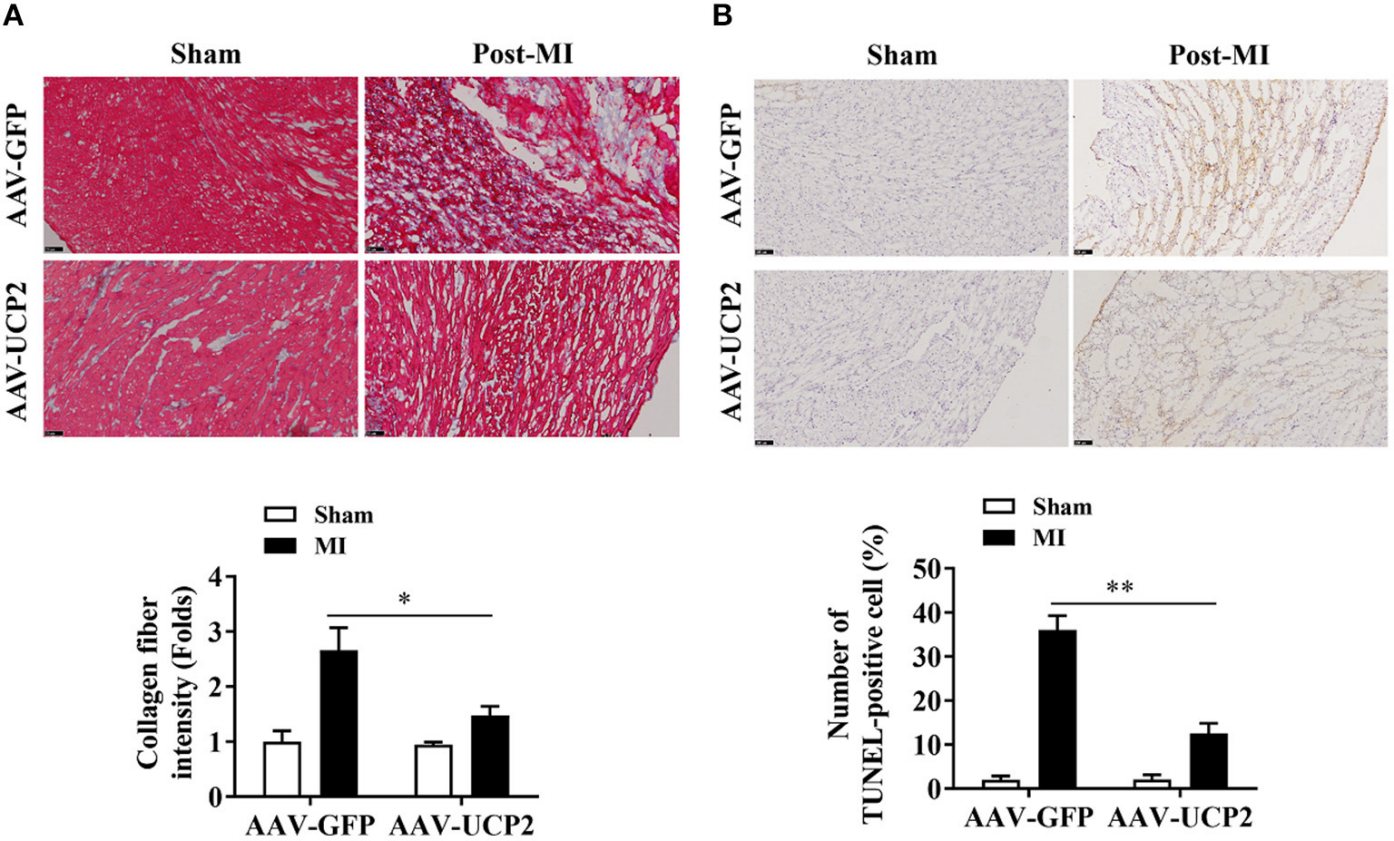
UCP2 overexpression in heart reduce myocardial fibrosis and cardiomyocyte apoptosis 4 weeks after MI. **(A)** Masson's trichrome staining of heart tissue sections showing the presence of myocardial fibrosis in the border zones of the infarcted regions 4weeks after MI (*n* = 3). **(B)** TUNEL assay showing the effects of overexpression UCP2 on cardiomyocyte apoptosis 4 weeks after MI (*n* = 3). MI, myocardial infarction; *p < 0.05, **P < 0.01 compared with each other.

To explore the role of UCP2 after MI, we investigated the effect of UCP2 on heart function after AAV-UCP2 transfection. The results showed that UCP2 significantly improved cardiac function. EF value increased from 40 to 55%, and other echocardiographic parameters (FS, LVIDd, LVIDs, IVSd, IVSs, LVPWd, and LVPWs) in the UCP2 group were also improved significantly (Figure 2). At the same time, we detected the level of fibrosis and found UCP2 reduced the intensity of collagen fiber (Figure 3A). In addition, we also detected apoptosis in different group. The TUNEL positive rate was 13% in the UCP2 group, while it was as high as 37% in the control group (Figure 3B).

### UCP2 Was a Direct Target Gene of miRNA-762

**Figure 4:**
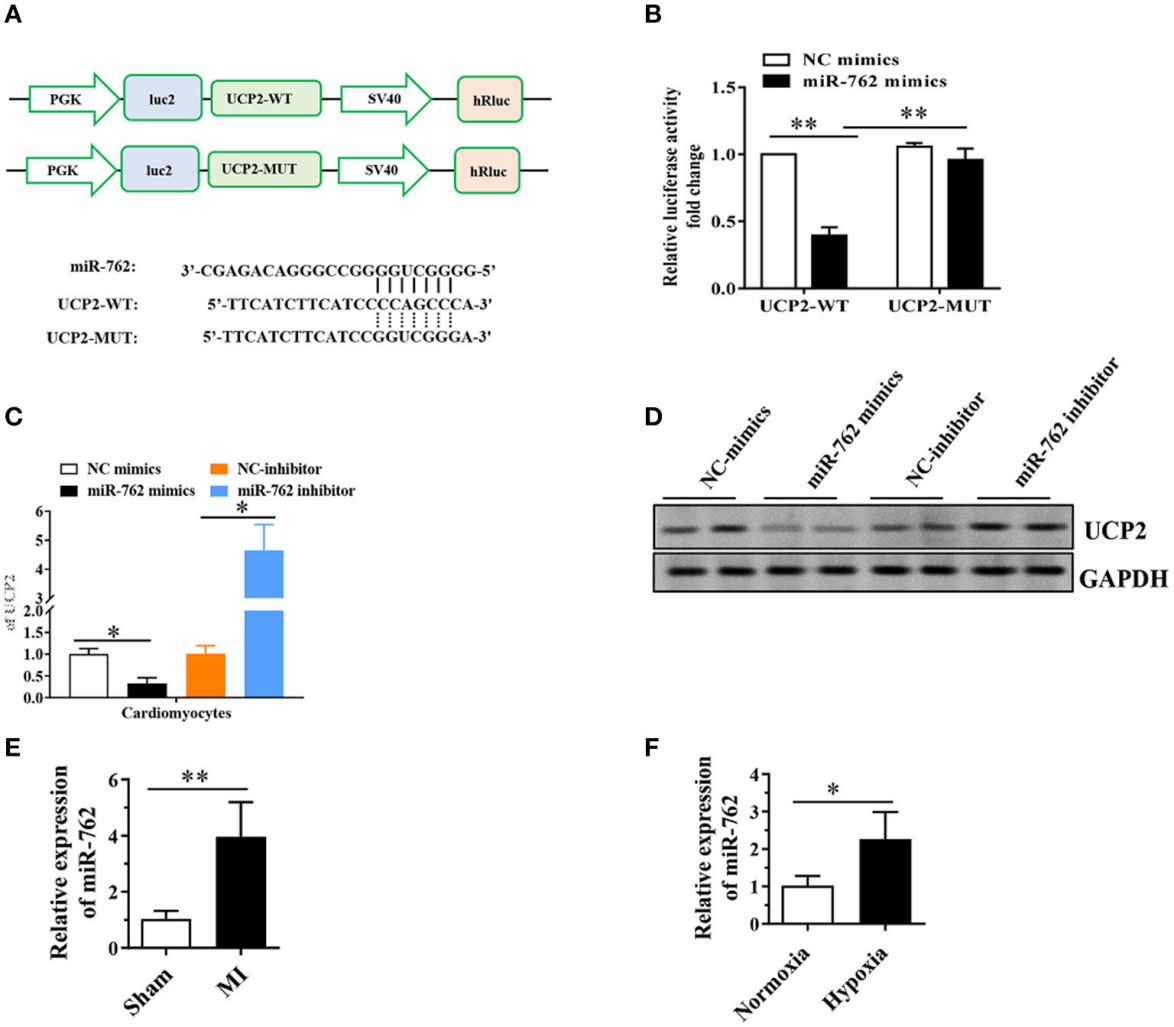
MiR-762 directly targets UCP2. **(A)** The putative miR-762 binding sequence and the mutant sequence in the 3′UTR of UCP2. **(B)** Luciferase reporter activities of vectors carrying luciferase gene and a fragment of UCP2 3′UTR containing the wild-type and mutant binding sites of miR-762. **(C)** Quantitative real-time PCR was performed to detect the expression of UCP2 mRNA. **(D)** Western blot was used to detect the level of UCP2 protein. **(E,F)** MiR-762 was significantly up-regulated in mouse myocardial tissue after MI and NMCMs cells subjected to hypoxia. The results were presented as the mean ± SD, *n* = 6, *P < 0.05. **P <0.01. MI, myocardial infarction.

To clarify the molecular mechanisms and underlying the role of UCP2 in cardiomyocytes, we used the TargetScan online tool to predict potential microRNA targets of UCP2 and discovered a conserved binding site for miR-762 in the 3′UTR region of the UCP2 gene. To confirm whether miR-762 targets UCP2 through its 3′ untranslated region (3′UTR), the wild-type or mutant 3′UTR fragment of UCP2 was cloned into a firefly luciferase reporter plasmid (Figure 4A). The luciferase activity assay showed that miR-762 attenuated the luciferase activity of the reporter containing the wild-type UCP2-3′UTR, but did not alter the activity of the reporter with the UCP2-3′UTR 3′UTR binding site mutation (Figure 4B). In addition, to verify that miR-762 targets UCP2 under physiological conditions, we have assessed the expression of UCP2 mRNA and protein in cells transfected with miR-762 mimics, mimic controls, miR-762 inhibitors, or inhibitor controls. Our results indicated miR-762 overexpression significantly lowered UCP2 mRNA and protein expression, while miR-762 inhibitor had little effect on UCP2 mRNA and protein expression (Figures 4C,D). These findings suggest that miR-762 can directly target UCP2 in NMCMs cells. In addition, quantitative real-time PCR was used to test the expression level of miR-762 in mouse myocardial tissues after MI and NMCMs cells subjected to hypoxia. As noted in Figures 4E,F, the level of miR-762 was notably increased in mouse myocardial tissues after MI and NMCMs cells subjected to hypoxia compared with control groups, respectively. Furthermore, we assessed whether manipulation of miR-762 levels could affect injury of NMCMs after hypoxia. These results indicate miR-762 reduce cell viability and increase cell damage after hypoxia; However,UCP2 overexpression could eliminate this effect of miR-762 on NMCMs (Supplementary Figure 1). These data suggest that UCP2 was a direct target gene of miRNA-762 and they have an important role in cell injury after hypoxia.

### The Effect of UCP2 on Fibrosis-Related Proteins and DRP1 Phosphorylation After MI

**Figure 5:**
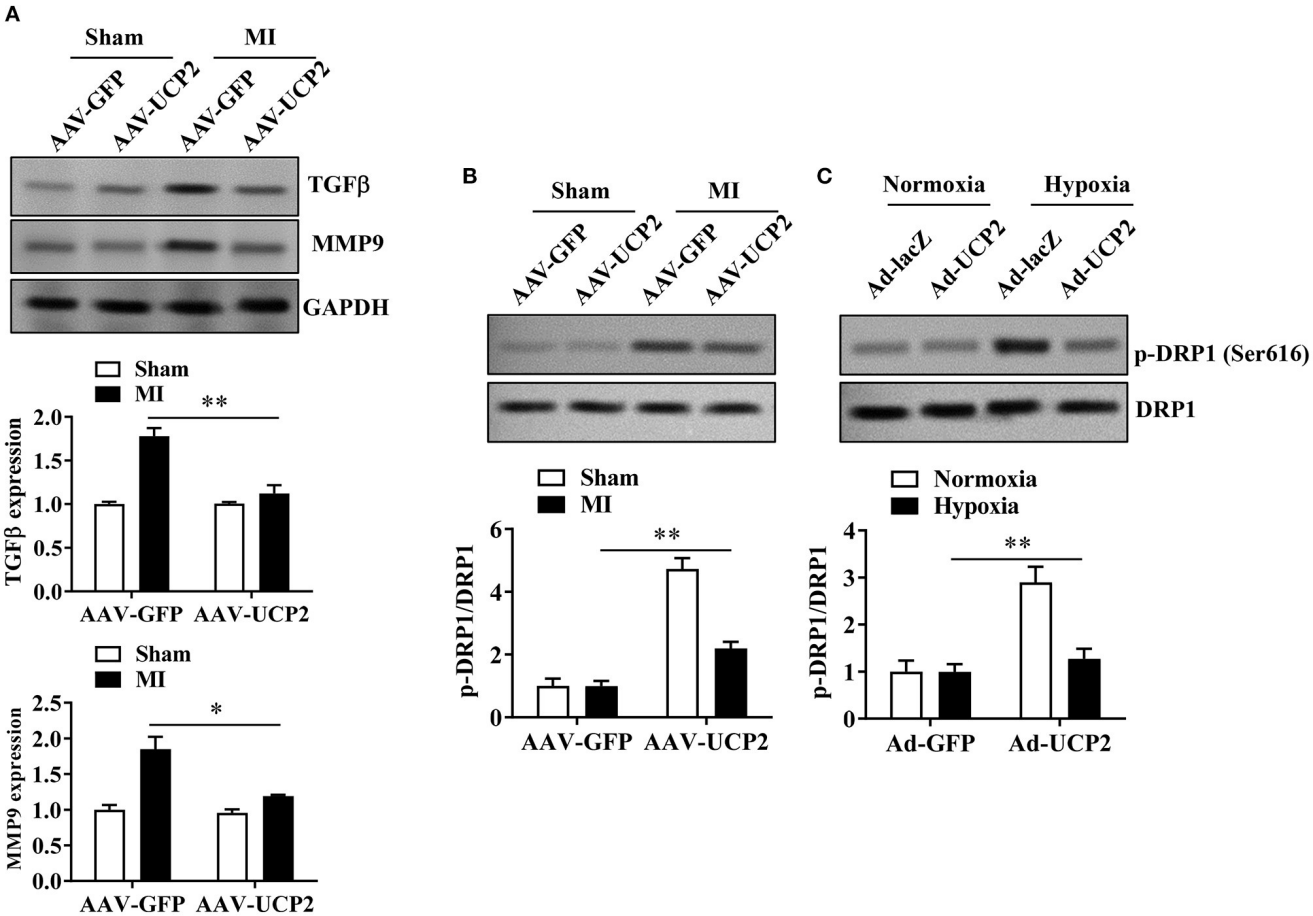
The effect of UCP2 overexpression on fibrosis-related proteins and DRP1 phosphorylation 4 weeks after MI. **(A)** UCP2 overexpression in heart reduces the expression of fibrosis-related protein MMP9 and TGF-β. **(B,C)** UCP2 overexpression in heart or cell increases the expression of *p*-DRP1/DRP1 4 weeks after MI. MI, myocardial infarction;*p < 0.05, **P < 0.01 compared with each other.

To explore the potential mechanism of UCP2 in alleviating myocardial fibrosis, we detected the expression of fibrosis-related proteins (MMP9 and TGF-β) by WB. We found that UCP2 reduced the expression of MMP9 and TGF-β (Figure 5A). A recent study showed that UCP2 is responsible for DRP1-dependent mitochondrial fission upon glucose load in SF1 neurons of ventromedial nucleus. Therefore, we infer UCP2 and DRP1 may have a similar interaction mechanism in cardiomyocytes. We have measured the level of DRP1 phosphorylation by WB *in vitro* and *in vivo*. It is found the phosphorylation of DRP1 and the ratio of p-DRP1/DRP1 decreased dramatically in the over-expressed UCP2 group (Figures 5B,C).

### UCP2 Confers Protection by Regulating DRP1 After Hypoxia

**Figure 6:**
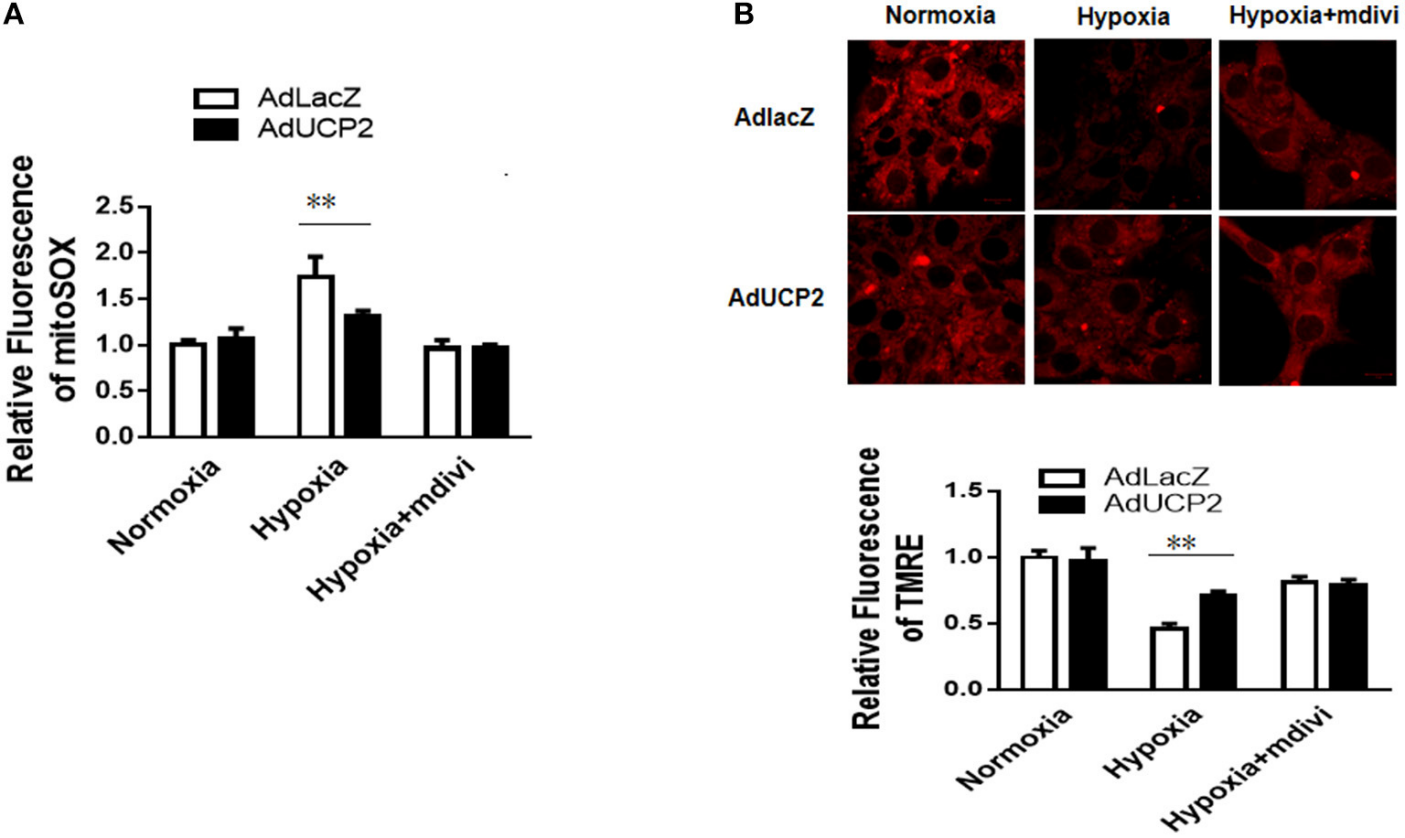
UCP2 affects the production of reactive oxygen species (ROS) and mitochondrial membrane potential (MMP) through DRP1. **(A)** The production of ROS was detected in different groups. **(B)** The MMP was measured in different groups. Mdivi, an inhibitor of DRP1; **P < 0.01 compared with each other.

**Figure 7:**
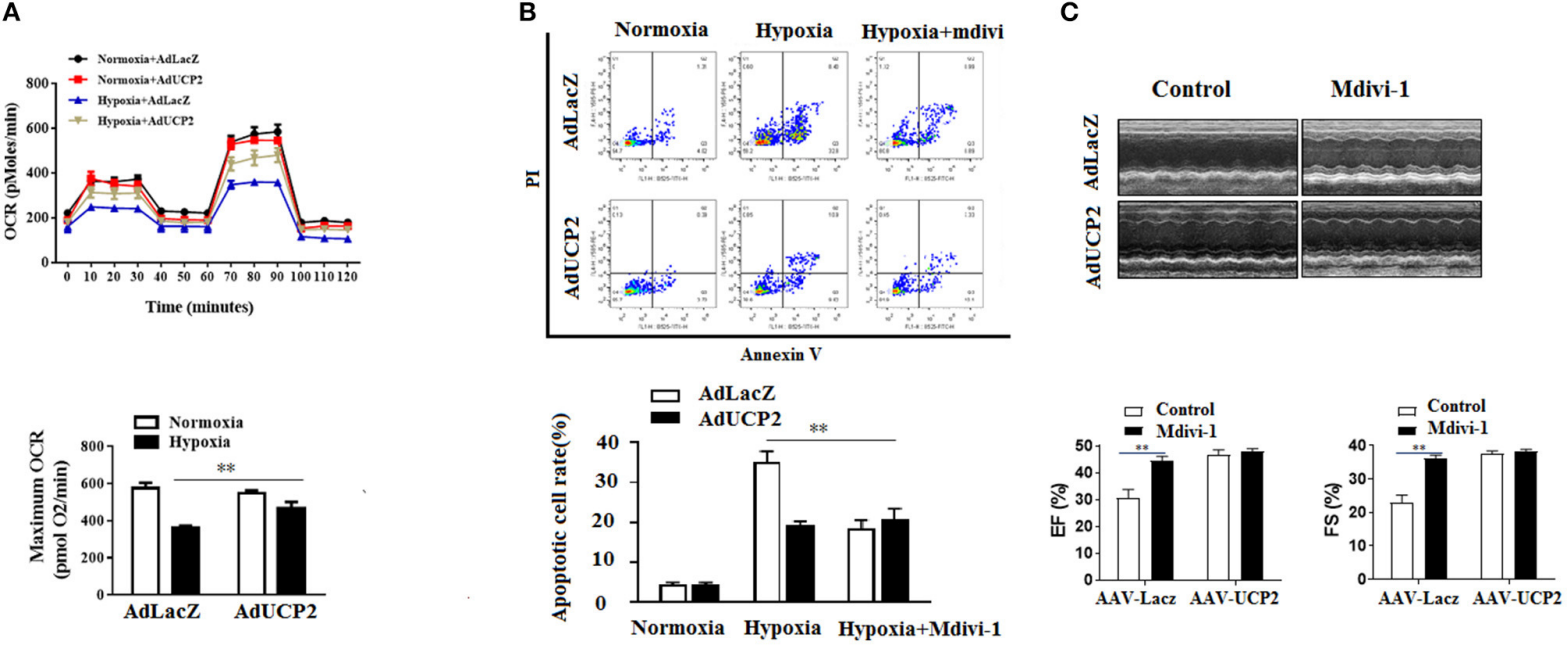
UCP2 affects cardiomyocyte oxygen consumption rate (OCR) and apoptosis through DRP1. **(A)** Cardiomyocyte OCR was measured in different groups. **(B)** Cardiomyocyte apoptosis was detected using flow cytometry in different groups. **(C)** M-mode images of the left ventricle (LV) in different groups were measured after MI. Mdivi, an inhibitor of DRP1; **P < 0.01 compared with each other.

It has been reported UCP2 affects mitochondrion fission and fusion to participate in regulating peripheral glucose homeostasis (20). Based on this, we infer that UCP2 may be involved in the process of cardiac remodeling after MI by affecting mitochondrial dynamic balance. Mdivi is widely used as a mitochondrial division inhibitor of DRP1. In this study, we used mdivi to inhibit DRP1 *in vitro* and *vivo*. As shown in Figure 6, the relative fluorescence of mitosox in mitochondria decreased significantly and the mitochondrial membrane potential was well-improved by mdivi. Furthermore, we detected OCR at different time points and found UCP2 significantly increased maximum OCR (Figure 7A). The effect of UCP2 on NMCMs' apoptosis and mouse cardiac function were detected by flow cytometry *in vitro* and M-mode echocardiography *in vivo*, respectively. It was shown UCP2 overexpression has a role in reducing apoptosis rate and improving cardiac function. However, compared with the mdivi group, inhibition of DRP1 by mdivi in over-expressed UCP2 group did not further reduce cell apoptosis rate and improve cardiac function, which indirectly indicates DRP1 is a key molecule for UCP2 to play its protective role after hypoxia and MI (Figures 7B,C).

## Discussion

Data presented in this study demonstrate: (1) The expression of UCP2 is downregulated in mouse heart 4 weeks after MI; (2) UCP2 overexpression in the heart has protective roles on myocardial fibrosis and apoptosis after MI; (3) The potential mechanism of UCP2 is involved in the microRNA-762 in upstream and DRP1-dependent mitochondrial fission downstream.

CHD remains the leading cause of death, accounting for 7.4 million deaths globally every year (21). Acute MI is a subtype of CHD, with high mortality and complicated pathophysiological process. To reduce mortality, it is necessary to explore the pathogenesis of the disease more deeply. UCP2 is one member of the family of mitochondrial anion carriers. In recent years, UCP2 was found to play an important role in heart disease. More and more evidence shows that the upregulation of UCP2 in the ischemic myocardium may be due to increased oxidative stress (17, 18). In this study, we have detected the expression of UCP2 4 weeks after MI with PCR and west blotting. Our results show that the expression of UCP2 decreased significantly,whichis in line with the previous study (22).

It was reported that UCP2 expression alters differently in the settings of coronary artery disease and myocardial ischemia. There is some evidence showing UCP2 reduces the CAD risk (12, 23). However, another study demonstrated the opposite result (13). The reason for this difference is not clear, but it may be due to race and population differences. In this study, we found UCP2 plays a positive role in failing hearts after MI. Overexpression of UCP2 in the heart after MI ameliorates heart failure and reduces myocardial fibrosis and apoptosis. Although UCP2 has an antioxidant effect on the myocardium, it also induces a low energy state of the myocardium through the uncoupling of oxidative phosphorylation. As a result, UCP2 has a potentially harmful effect on failing hearts, with mismatches in energy production and utilization (22). However, in this study, it is found proper external supply of UCP2 protected against heart failure after MI. We speculate the possible reason is that UCP2 keeps the heart after MI in a good state of energy balance.

In the present study, we explored the potential protective mechanism of UCP2 after MI. Although the exact mechanism is not completely clear, some authors believe that UCP2 affects many aspects of mitochondrial function. It is reported that UCP2 prevents cell death caused by mitochondria (18). In addition, recent research has demonstrated UCP2 takes responsibility for DRP1-dependent mitochondrial fission upon glucose load in the ventromedial nucleus' SF1 neurons. Therefore, we focused on exploring the relationship between UCP2 and DRP1 after MI. Not surprisingly, we found UCP2 plays its protective role by regulating DRP1-dependent mitochondrial function and cardiomyocyte apoptosis. DRP1 is the most studied fission protein in heart disease, whose sub-pool colocalizes with mitochondria at sites of future fission. mitochondrial fission induced by DRP1 is an important mediator of myocardial cell death in MI (14, 24). The inhibition of DRP1 also protects the heart from MI by reducing mitochondrial metabolism and fragmentation (25). It was indicated in our study that UCP2 affected the production of mitochondrial superoxide, mitochondrial membrane potential, mitochondrial oxygen consumption rate, and cardiomyocyte apoptosis and improve cardiac function by DRP1. DRP1 is essential for UCP2 to play its protective role after MI.

MicroRNAs (miRNAs) are a big family of small non-coding RNAs that regulate gene expression by binding to their target mRNAs, subsequently leading to translation inhibition or degradation. Numerous studies have shown that miRNAs are involved in various heart diseases such as hypertension, hypertrophy, and remodeling. This study explored the relationship between miRNA and UCP2 after MI. we used bioinformatics analysis, luciferase activity and western blot assay proved UCP2 was a direct target gene of miRNA-762. Previous study show miR-762 participates in the regulation of cardiomyocyte apoptosis and mitochondrial function by NADH dehydrogenase subunit 2 (ND2) (26). In our study, we found miR-762 reduce cell viability and increase cell damage after hypoxia and it plays the role by the target of UCP2. MiR-762 and UCP2 may be new therapeutic targets for patients after MI.

### Limitations

However, it should be noted that there are still some shortcomings in this study. Firstly, the study did not use transgenic mice to explore the role and mechanism of UCP2. Secondly, miRNA microarray assay isn't employed to explore the relationship between UCP2 and miRNAs.we just use targetscan software to predict the target of UCP2 and verify the result by luciferase activity and western blot.

In summary, our study revealed the physiological role of UCP2 in mouse heart after MI. The potential protective mechanism of UCP2 is involved in miRNA-762 upstream and DRP1-dependent mitochondrial fission downstream. UCP2 may offer new therapeutic potential for patients after MI.

## Data Availability Statement

The original contributions presented in the study are included in the article/Supplementary Materials, further inquiries can be directed to the corresponding author/s.

## Ethics Statement

The animal study was reviewed and approved by Guangdong Provincial People's Hospital.

## Author Contributions

YL: conceptualization, design, and writing. DL: data curation, writing-original draft preparation, and software. SC: visualization. CC: investigation. SZ: supervision. HS and JW: methodology. QZ and CL: validation. YW: writing—reviewing and editing. GL: design and supervision. All authors contributed to the article and approved the submitted version.

## Funding

This work was partly supported by the Key Project of Natural Science Foundation of Guangdong Province (2017B030311010).

## Conflict of Interest

The authors declare that the research was conducted in the absence of any commercial or financial relationships that could be construed as a potential conflict of interest.

## Publisher's Note

All claims expressed in this article are solely those of the authors and do not necessarily represent those of their affiliated organizations, or those of the publisher, the editors and the reviewers. Any product that may be evaluated in this article, or claim that may be made by its manufacturer, is not guaranteed or endorsed by the publisher.

## Supplementary Material

Supplementary Figure 1 The effect of MiR-762 and UCP2 on cell viability and damage. **(A)** Cell viability was measured by MTT assay. **(B)** LDH was measured using LDH assay kit **P* < 0.05 compared with each other.

## Abbreviations

CHD: Coronary heart disease
DRP1: Dynamin-related protein1
DMEM: Dulbecco's modified eagle medium
EF: Ejection fraction
FS: Fractional shortening
FBS: Fetal bovine serum
IVSs: Interventricular septal systolic diameter
IVSd: Interventricular septal diastolic diameter
LAD: Left anterior descending
LVIDd: Left ventricular end diastolic diameter
LVIDs: Left ventricular end systolic diameter
LVPWd: Left ventricular posterior wall diastolic diameter
LVPWs: Left ventricular posterior wall systolic diameter
MMP9: Matrix metallo proteinase 9
MI: Myocardial infarction
NMCMs: Neonatal mouse cardiomyocytes
PBS: Phosphate buffer saline
ROS: Reactive oxygen species
RT-PCR: Reverse transcription-quantitative polymerase chain reaction
TGF-β: Transforming growth factor-beta
TUNEL: Transferase-mediated dUTP nick end-labeling
UCP2: Uncoupling protein 2.
