# Supplementary figures and images for: Downregulation of Uncoupling Protein 2(UCP2) Mediated by MicroRNA-762 Confers Cardioprotection and Participates in the Regulation of Dynamic Mitochondrial Homeostasis of Dynamin Related Protein1 (DRP1) After Myocardial Infarction in Mice
Source: Front Cardiovasc Med. 2022 Feb 24;8:764064. doi: 10.3389/fcvm.2021.764064 (PMC8907155; doi:10.3389/fcvm.2021.764064)

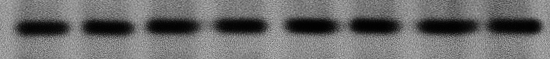

Supplement: Supplementary file 4 [file Data_Sheet_4.zip › Figure4/GAPDH.tif]

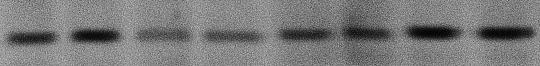

Supplement: Supplementary file 4 [file Data_Sheet_4.zip › Figure4/UCP2.tif]

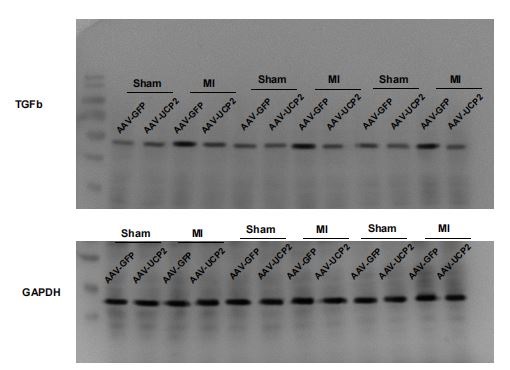

Supplement: Supplementary file 5 [file Data_Sheet_5.zip › Figure5/Figure 5A.TGFb and MMP9 wb/Figure 5a.JPG]

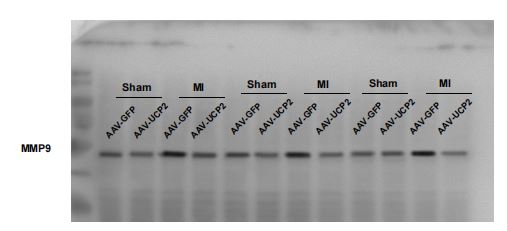

Supplement: Supplementary file 5 [file Data_Sheet_5.zip › Figure5/Figure 5A.TGFb and MMP9 wb/Figure 5a2.JPG]

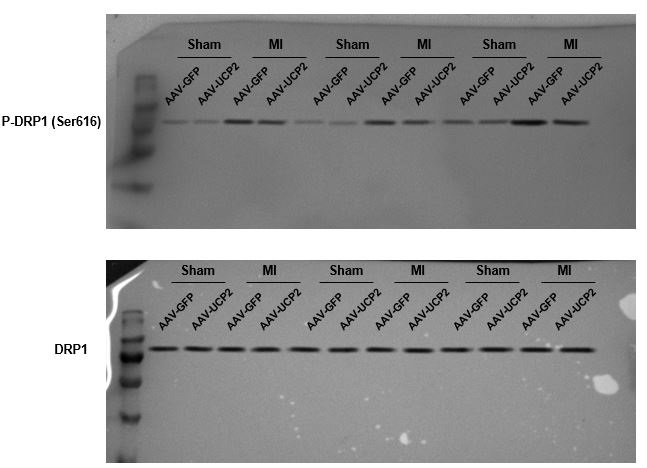

Supplement: Supplementary file 5 [file Data_Sheet_5.zip › Figure5/Figure 5BC.DRP-1/Figure 5b.JPG]

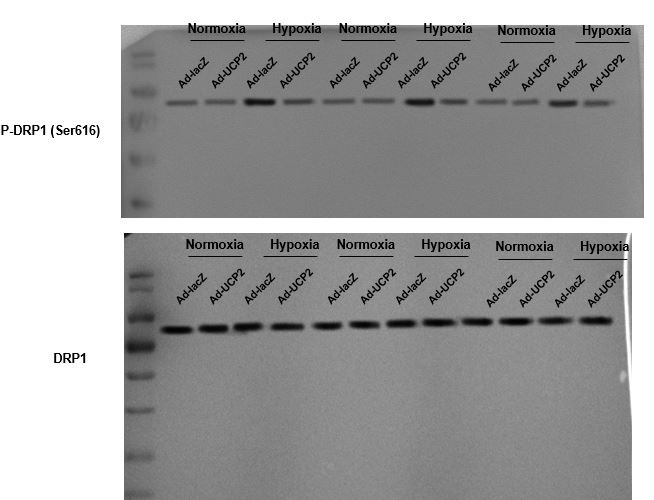

Supplement: Supplementary file 5 [file Data_Sheet_5.zip › Figure5/Figure 5BC.DRP-1/Figure 5c.JPG]

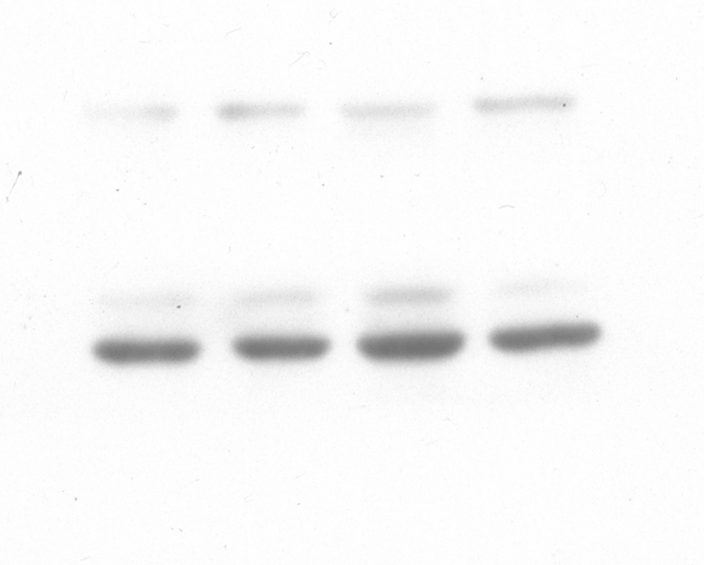

Supplement: Supplementary file 5 [file Data_Sheet_5.zip › Figure5/Figure 5BC.DRP-1/cell/DRP1-ori.tif]

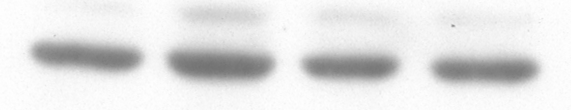

Supplement: Supplementary file 5 [file Data_Sheet_5.zip › Figure5/Figure 5BC.DRP-1/cell/DRP1.tif]

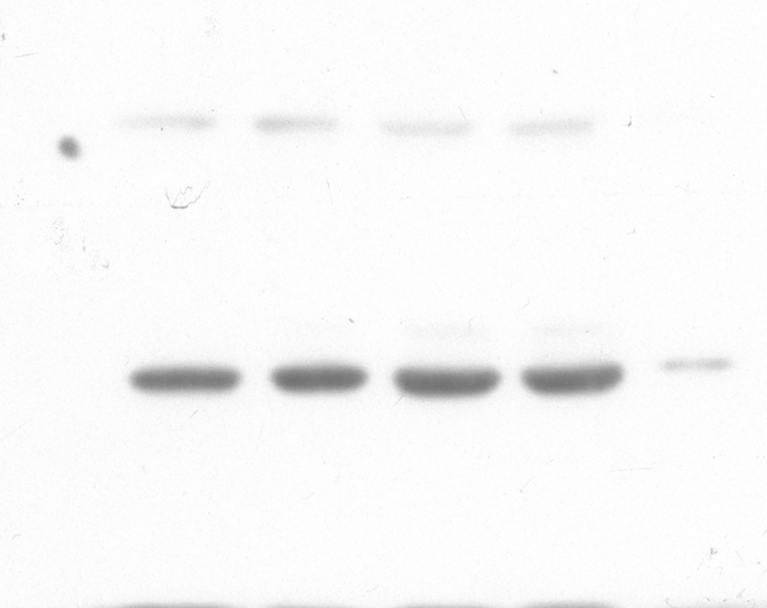

Supplement: Supplementary file 5 [file Data_Sheet_5.zip › Figure5/Figure 5BC.DRP-1/cell/GAPDH-ori.tif]

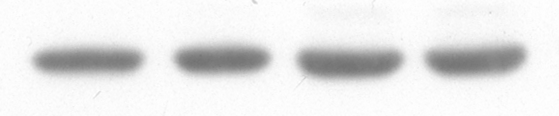

Supplement: Supplementary file 5 [file Data_Sheet_5.zip › Figure5/Figure 5BC.DRP-1/cell/GAPDH.tif]

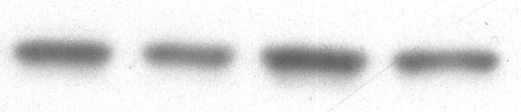

Supplement: Supplementary file 5 [file Data_Sheet_5.zip › Figure5/Figure 5BC.DRP-1/cell/p-DRP1.tif]

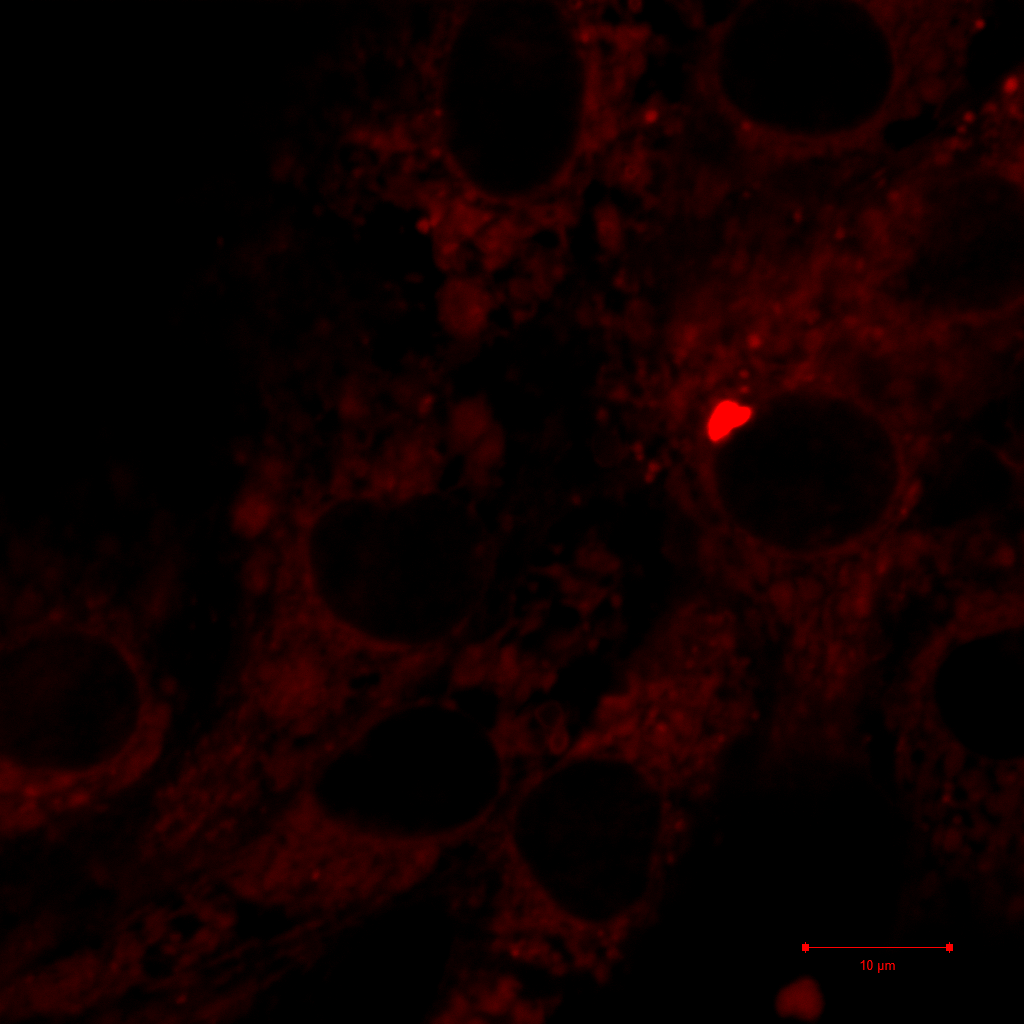

Supplement: Supplementary file 6 [file Data_Sheet_6.zip › Figure6/13-AdlacZ-Hypoxia.tif]

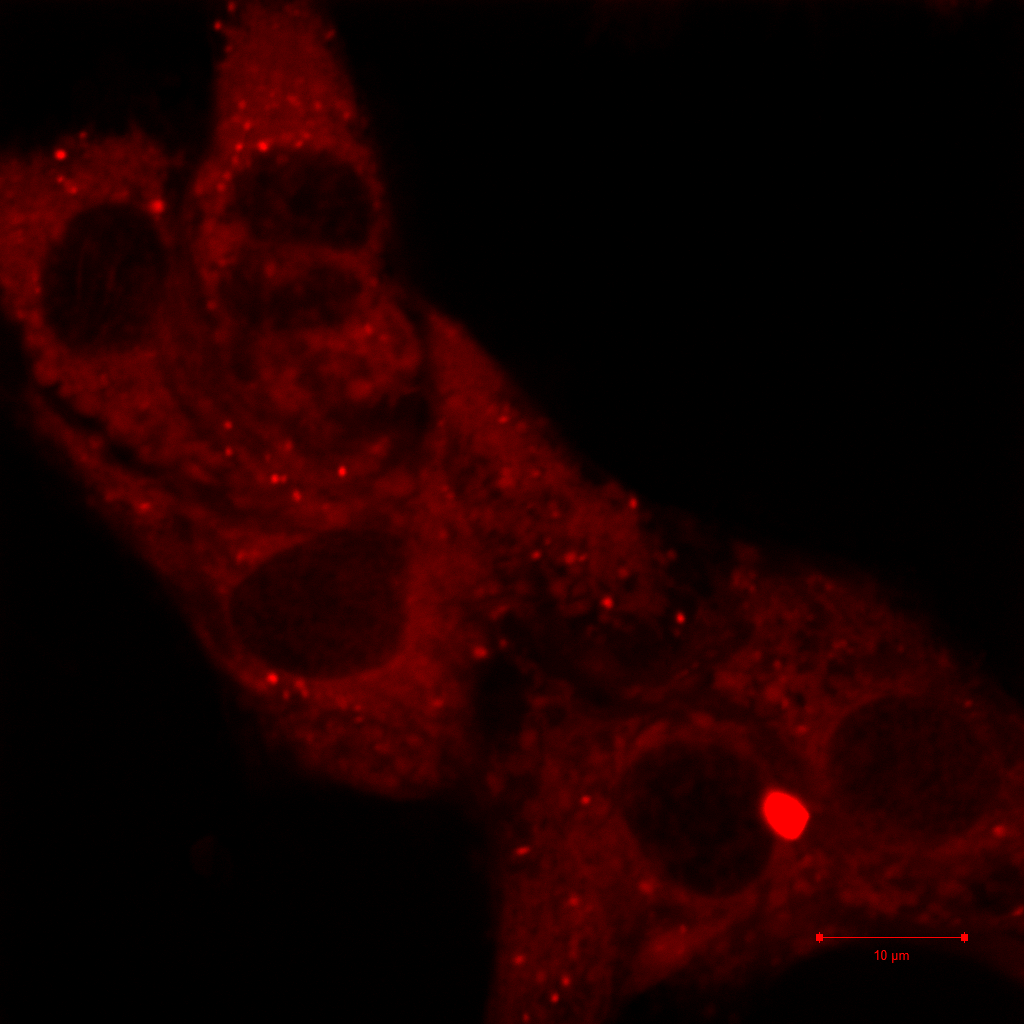

Supplement: Supplementary file 6 [file Data_Sheet_6.zip › Figure6/13-lacZ-mdivi-hypoxia.tif]

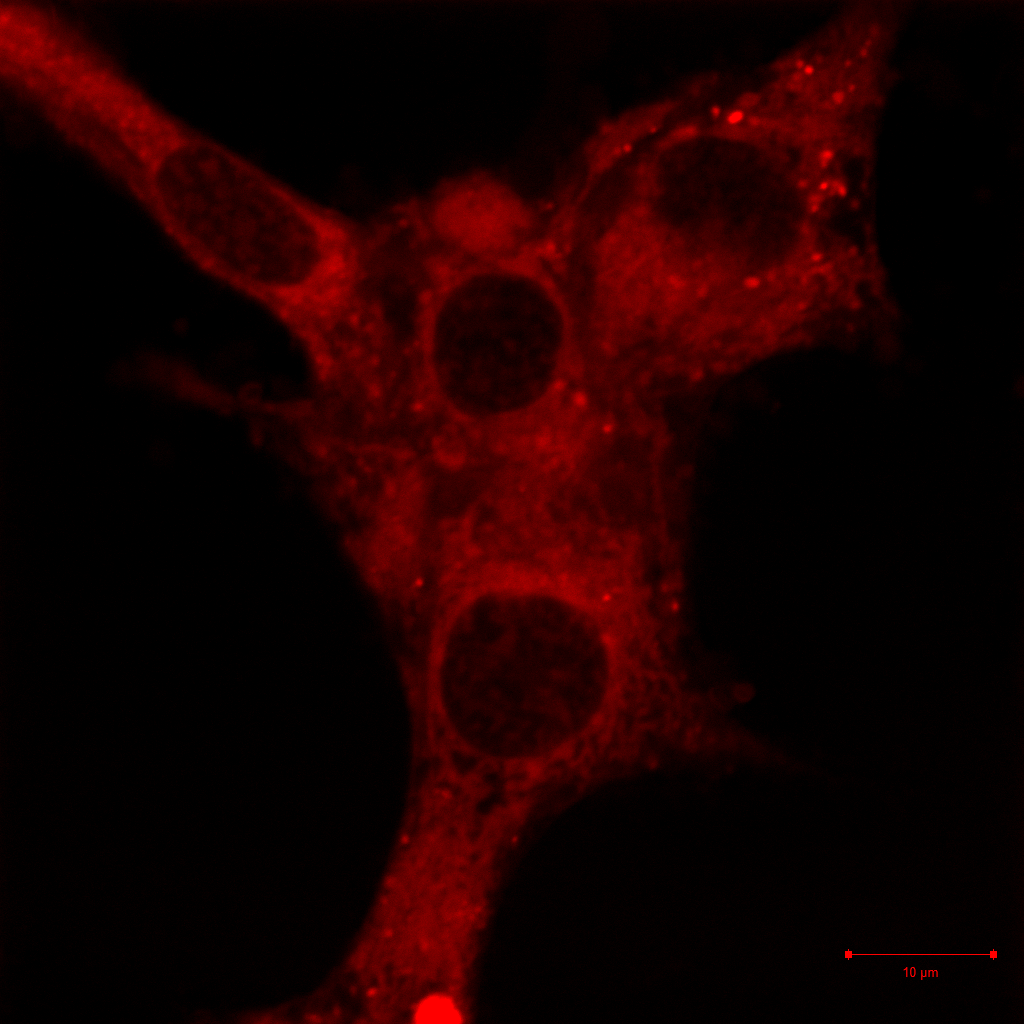

Supplement: Supplementary file 6 [file Data_Sheet_6.zip › Figure6/14-AdUCP2-mdivi-hypoxia.tif]

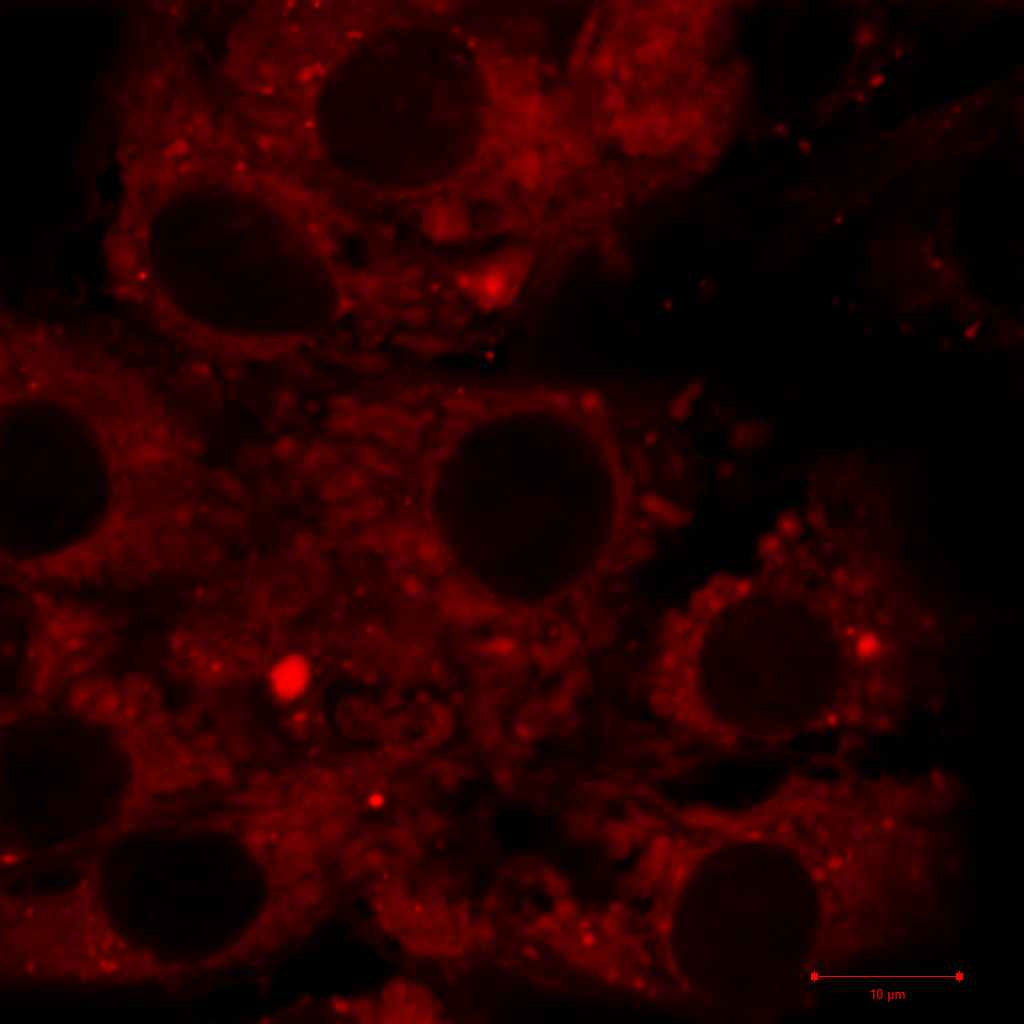

Supplement: Supplementary file 6 [file Data_Sheet_6.zip › Figure6/22-AdUCP2-Hypoxia.tif]

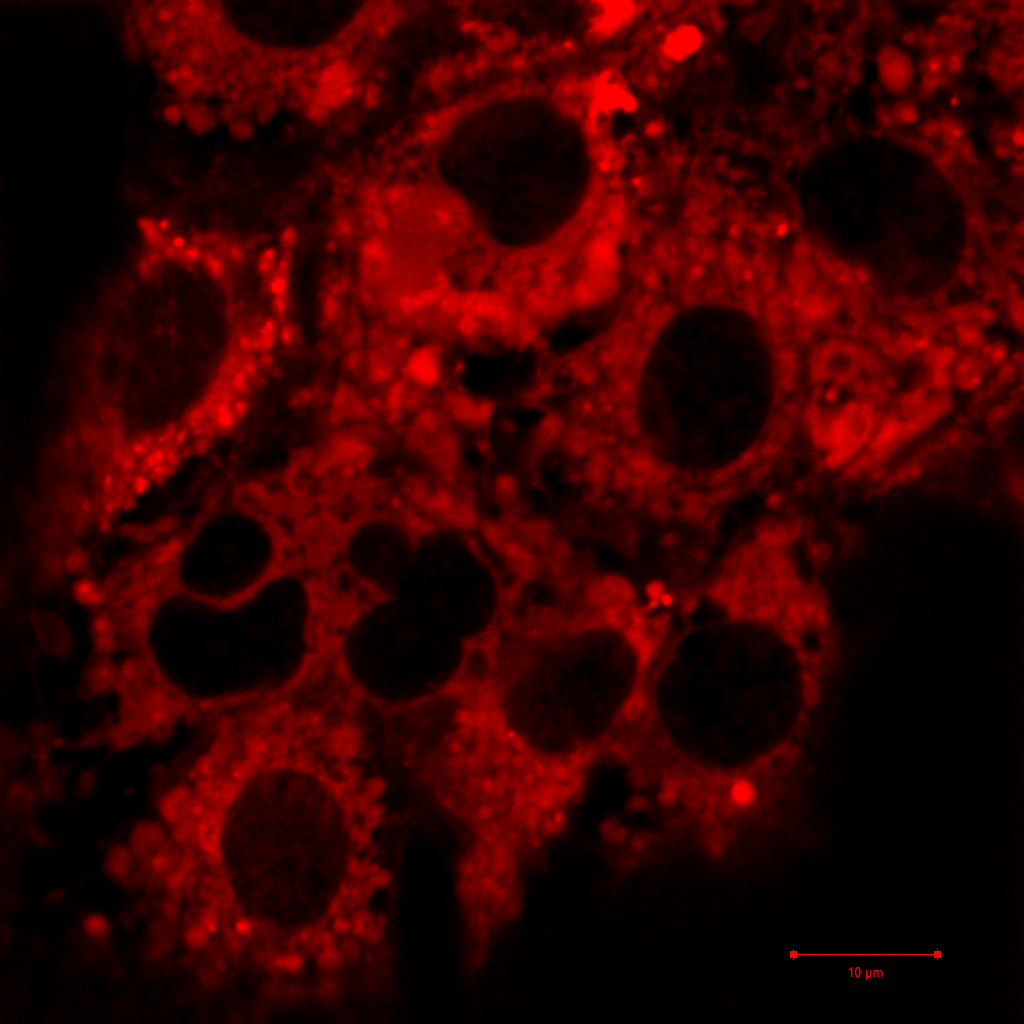

Supplement: Supplementary file 6 [file Data_Sheet_6.zip › Figure6/4-AdlacZ-Nor.tif]

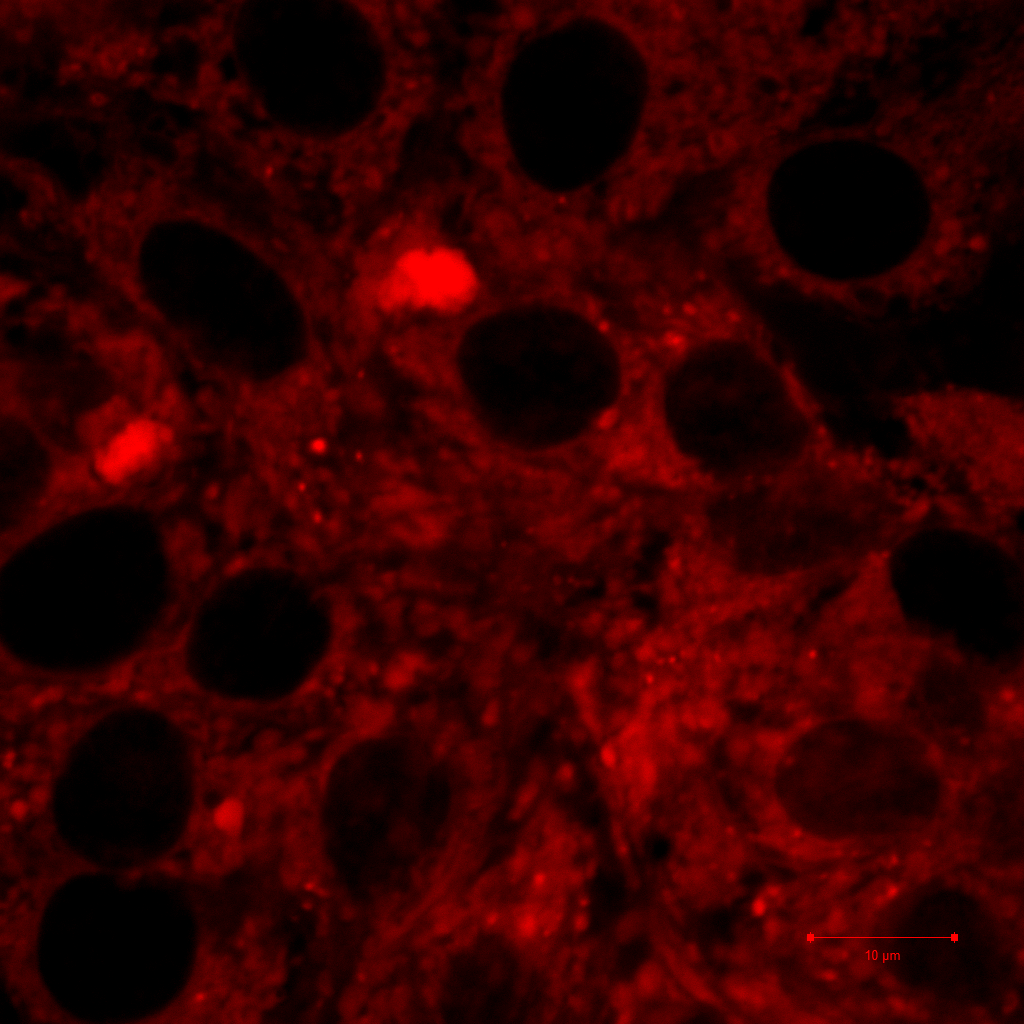

Supplement: Supplementary file 6 [file Data_Sheet_6.zip › Figure6/6-AdUCP2-Nor.tif]
